# p62/Sequestosome 1 Regulates Aggresome Formation of Pathogenic Ataxin-3 with Expanded Polyglutamine

**DOI:** 10.3390/ijms150914997

**Published:** 2014-08-25

**Authors:** Liang Zhou, Hongfeng Wang, Dong Chen, Feng Gao, Zheng Ying, Guanghui Wang

**Affiliations:** 1 Laboratory of Molecular Neuropathology, Jiangsu Key Laboratory of Translational Research and Therapy for Neuro-Psycho-Diseases and College of Pharmaceutical Sciences, Soochow University, Suzhou 215021, China; E-Mails: zl123@mail.ustc.edu.cn (L.Z.); wanghongfeng@suda.edu.cn (H.W.); chendong@suda.edu.cn (D.C.); fgao@suda.edu.cn (F.G.); 2 Key Laboratory of Brain Function and Disease, School of Life Sciences, University of Science & Technology of China, Chinese Academy of Sciences, Hefei 230027, China

**Keywords:** ataxin-3, polyglutamine, p62, aggregation, aggresome

## Abstract

The cellular protein quality control system in association with aggresome formation contributes to protecting cells against aggregation-prone protein-induced toxicity. p62/Sequestosome 1 (p62) is a multifunctional protein which plays an important role in protein degradation and aggregation. Although poly-ubiquitination is usually required for p62-mediated protein degradation and aggresome formation, several p62 substrates are processed to form aggregate in an ubiquitination-independent manner. In this study we demonstrate that p62 directly interacts with pathogenic Machado Joseph Disease (MJD)-associated protein ataxin-3 with polyglutamine (polyQ) expansion. Moreover, p62 could regulate the aggresome formation of pathogenic ataxin-3 and protect cells against pathogenic ataxin-3-induced cell death.

## 1. Introduction

Machado Joseph Disease (MJD)/Spinocerebellar ataxia type 3 (SCA3), the most common SCA subtype, is a polyglutamine disease caused by the expansion of a CAG stretch in *MJD1* gene [1,2]. The *MJD1* gene in unaffected persons contains 12–40 CAG repeats, whereas mutant *MJD1* gene in patients has 62–86 CAG repeats [1]. The CAG repeat expansion in the MJD1 gene results in an expanded polyglutamine (polyQ) tract in the encoded protein ataxin-3 [3].

Normal ataxin-3 is diffusively distributed in whole cell, but the pathogenic (polyQ expanded) ataxin-3 tends to form aggregate [4]. Protein aggregates have also been found in other polyQ diseases, such as Huntington’s disease and SCA1 [5,6,7]. The polyQ-containing aggregates are found in the nucleus [4,8], cytoplasm [9] and axon [10], and protein aggregation is a neuropathological hallmark of MJD and many other neurodegenerative diseases [11,12]. It has been reported that the aggregates can be transported along microtubule towards microtubule organizing center (MTOC) to generate a large inclusion structure named aggresome. This process is usually thought to protect cells against the aggregated protein-induced cell death [13,14,15], and aggresomes formed by pathological proteins have been broadly discovered in neurodegenerative diseases, such as synphilin-1 and DJ-1 aggresomes in Parkinson’s disease, huntingtin aggresomes in Huntington’s disease and copper-zinc superoxide dismutase (SOD1) aggresomes in amyotrophic lateral sclerosis (ALS), respectively [15,16,17,18,19,20,21]. However, up to date, it is still unknown whether pathogenic ataxin-3 could form aggresomes.

p62/Sequestosome1 (p62), mutation of which is associated with human disorders such as ALS and Paget disease of the bone, is a common component of protein aggregates and aggresomes in neurodegenerative disorders [22,23,24]. p62 interacts with poly-ubiquitinated proteins and microtubule-associated protein 1 light chain 3 (LC3) to function in autophagy-lysosome pathway, and p62 also functions in protein aggregate and aggresome formation [23,24,25,26]. Although many misfolded proteins undergo poly-ubiquitination, which is a signal for recognition by p62 and for aggregate formation, several interesting studies showed that p62 substrates could be processed to form aggregate in an ubiquitination independent manner. For example, p62 regulates mutant SOD1 aggregation by directly interacting with mutant SOD1 [22,23,27].

In the current study we show that ataxin-3-Q80, a type of pathogenic ataxin-3 with polyQ expansion, forms aggresome under proteasome dysfunction in cultured cells. We also found that p62 can regulate aggresome formation of pathogenic ataxin-3, and p62 physically interacts with pathogenic ataxin-3, but not normal ataxin-3. Moreover, p62 regulates the aggresome formation of polyQ expanded ataxin-3 in a microtubule dependent manner, and protects cells against the polyQ expanded ataxin-3-induced cell death.

## 2. Results and Discussion

### 2.1. p62 Directly Interacts with Ataxin-3-Q80

**Figure 1:**
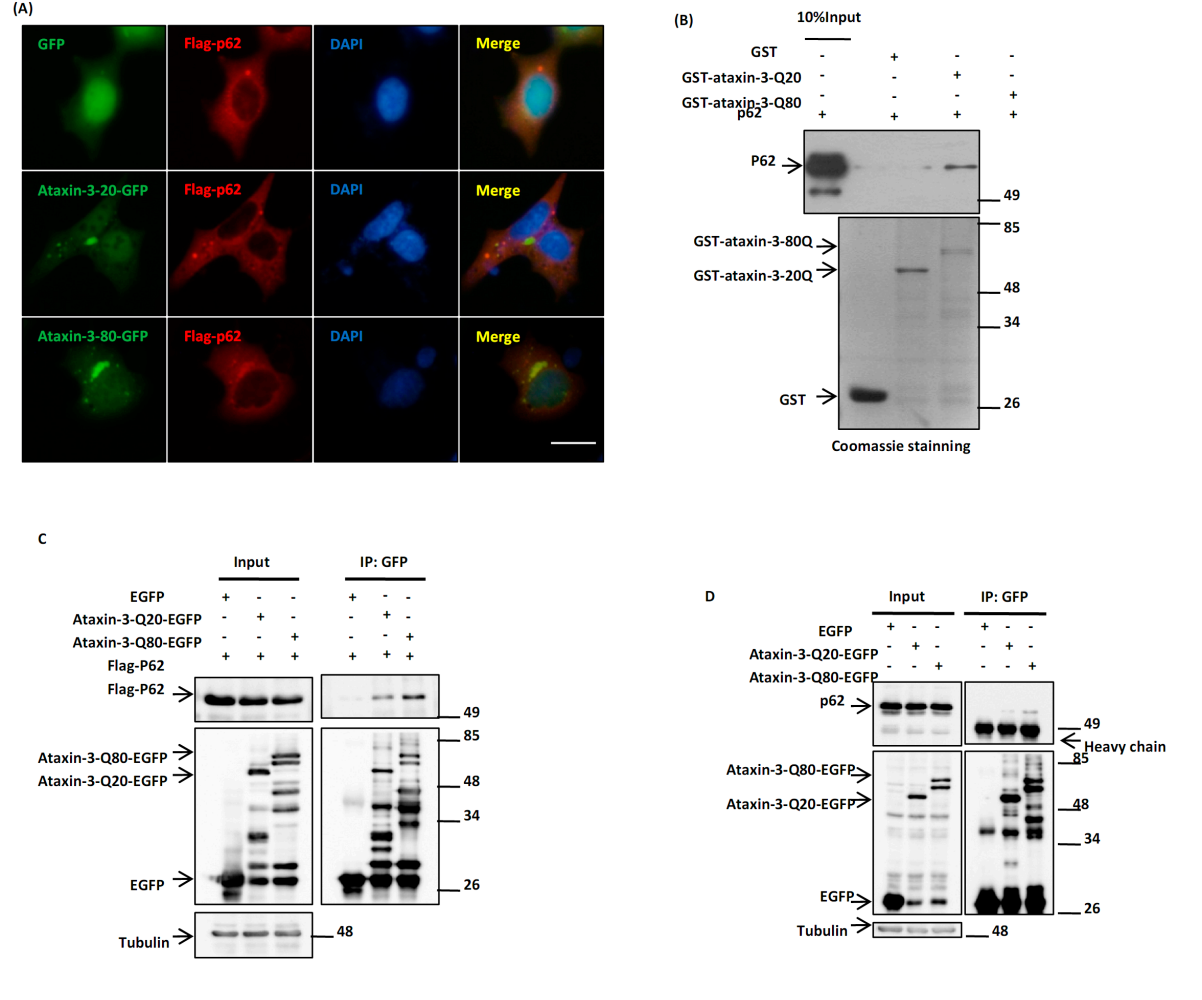
p62 directly interacts with ataxin-3-Q80. (**A**) Ataxin-3-Q80 co-localizes with p62. Human embryonic kidney 293 (HEK293) cells were co-transfected with Flag-p62 and EGFP, ataxin-3-Q20-EGFP or ataxin-3-Q80-EGFP. After 48 h, the cells were fixed and subjected to immunofluorescent assay with an anti-Flag antibody (red). The cell nuclei were stained with DAPI. The scale bar indicates 10 µm; (**B**) p62 directly interacts with ataxin-3-Q80. The GST pulldown assay was performed using purified GST, GST-ataxin-3-Q20 or GST-ataxin-3-Q80 to be incubated with p62, and then the precipitants were subjected to immunoblot analysis with anti-p62 and anti-GST antibodies; (**C**) Flag-p62 and EGFP, ataxin-3-Q20-EGFP or ataxin-3-Q80-EGFP were co-transfected into 293 cells for 48 h, and the cell lysates were immunoprecipitated with an anti-GFP antibody. Then the immunoprecipitants were subjected to immunoblot analysis with the anti-Flag-HRP or anti-tubulin antibodies; (**D**) H1299 cells were transfected with EGFP, ataxin-3-Q20-EGFP or ataxin-3-Q80-EGFP for 48 h, and then the immunoprecipitation assay was performed with an anti-GFP antibody.

The aggregation of polyQ expanded ataxin-3 is a neuropathological hallmark of MJD [11,12], and p62 is known to be recruited to those ataxin-3 inclusions in MJD patient brains [10]. In culture cells, we found that the pathogenic ataxin-3 with polyQ expansion (ataxin-3-Q80), but not normal ataxin-3 (ataxin-3-Q20), was specifically co-localized with p62 (Figure 1A). To investigate the molecular mechanism underlying this phenomenon, we tested whether there is a direct interaction between p62 and ataxin-3. *In vitro* pulldown assay showed that ataxin-3-Q80, but not ataxin-3-Q20, directly interacted with p62 (Figure 1B). Moreover, immunoprecipitation assay showed that ataxin-3-Q80 exhibited a higher affinity with both exogenous and endogenous p62 than ataxin-3-Q20 did (Figure 1C,D). These data indicated that p62 may play a direct role in the regulation of ataxin-3-Q80.

### 2.2. p62 Promotes the Aggresome Formation of Ataxin-3-Q80

**Figure 2:**
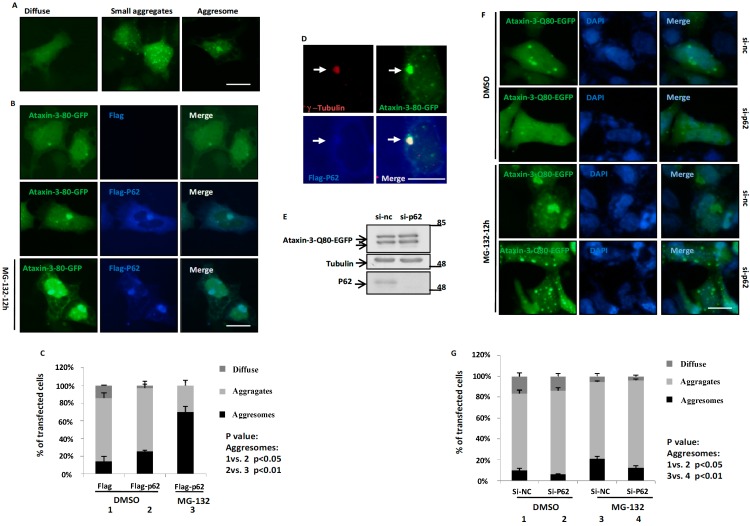
p62 promotes the aggresome formation of ataxin-3-Q80. (**A**) The three distinct distribution patterns of ataxin-3-Q80-EGFP in HEK293 cells. Ataxin-3-Q80-EGFP was transfected into HEK293 cells for 48 h, and then visualized using an invert fluorescent microscope. The scale bar indicates 10 µm; (**B**) Ataxin-3-Q80-EGFP and Flag or Flag-p62 were transfected into the HEK293 cells for 48 h, and then treated with DMSO or MG-132 (10 µM) for another 12 h. Finally, the cells were fixed and subjected to immunofluorescent assay with anti-Flag antibody (blue). The scale bar indicates 10 µm; (**C**) The three distinct distribution patterns of ataxin-3-Q80-EGFP in (**B**) are quantified. Three independent experiments were performed and the data are indicated as the means ± S.E.M; (**D**) HEK293 cells were transfected with EGFP-ataxin-3-80Q and FLAG-p62 for 48 h, and then treated with MG132 (10 µM) for another 12 h. Subsequently, the cells were subjected to immunofluorescent assay using anti-γ-tubulin (Red) and p62 (blue) antibodies. The scale bar indicates 10 µm; (**E**) Knockdown of p62 in HEK293 cells. Transfect the si-nc or si-p62 siRNA (1# plus 2#) into HEK293 cells for 72 h, the cell lysates were subjected to immunoblot analysis using anti-p62 and anti-tubulin antibodies; (**F**) HEK293 cells were transfected with si-p62 (1# plus 2#) or si-nc siRNA. After 12 h, cells were transfected with ataxin-3-Q80-EGFP for 48 h, and then treated with DMSO or MG-132 (10 µM) for 12 h. At last, the cells were fixed and subjected to immunofluorescent assay at 72 h. The cells nuclei were stained with DAPI. The scale bar indicates 10 µm; (**G**) The three distinct distribution patterns of ataxin-3-Q80-EGFP in (**F**) are quantified. Three independent experiments were performed and the data are indicated as the means ± S.E.M.

As p62 can regulate the protein aggregation of mutant huntingtin and SOD1 [23,25] to play a protective role, we wondered whether p62 plays a role in the aggregation of ataxin-3-Q80. In cultured cell model, ataxin-3-Q80 displayed three distinct distribution forms: (1) diffusion throughout the cells; (2) multiple small aggregates; (3) large inclusions (Figure 2A). As the large inclusions were singularity and juxtanuclear (Figure 2A,B,F), and the inclusion formation depended on microtubule integrity (Figure 4B,C), we speculated that they are aggresomes. As predicted, results confirmed that those inclusions formed by ataxin-3-80Q and p62 colocalized with γ-tubulin, an MTOC and aggresome marker (Figure 2D). When we used MG-132, a proteasome inhibitor, the rate of aggresome formation was strikingly increased in cells (Figure 2B). Overexpression of p62 promoted the aggresome formation of ataxin-3-Q80 (Figure 2B,C), whereas knockdown of p62 inhibited the aggresome formation of ataxin-3-Q80 under both normal or MG-132 treated condition (Figure 2D–F). These data suggest that p62 can promote the aggresome formation of ataxin-3-Q80.

### 2.3. p62 Has no Effect on the Protein Expression of Ataxin-3

**Figure 3:**
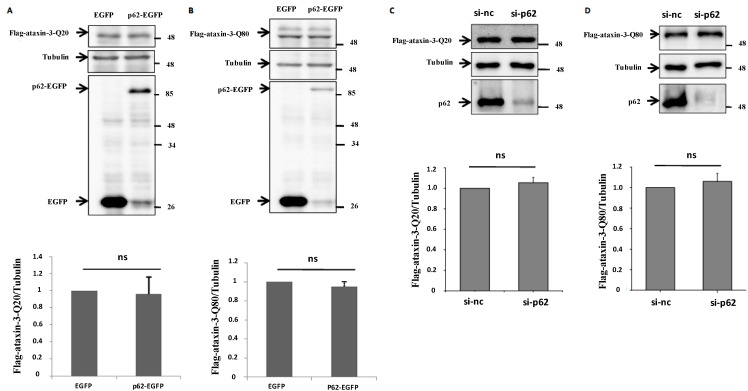
p62 has no affect on the protein expression of ataxin-3-Q20/Q80. (**A**,**B**) HeLa cells were transfected with Flag-ataxin-3-Q20/Q80 for 12 h, then transfected with p62-EGFP for another 48 h. The cell lysates were subjected to immunoblot analysis with anti-Flag-HRP, anti-tubulin, anti-GFP antibodies. The relative density ratios of the Flag-ataxin-3-Q20/Q80 to tubulin were determined. The data are indicated as the means ± S.E.M. ns, no significance; (**C**,**D**) HeLa cells were transfected with si-nc or si-p62 siRNA(1# plus 2#) for 12 h, then transfected with Flag-ataxin-3-Q20/Q80 for another 48 h. The cell lysates were subjected to immunoblot analysis with anti-Flag-HRP, anti-tubulin, anti-p62 antibodies. The quantitative data are indicated as the means ± S.E.M. ns, no significance.

Given that p62 is a key regulator in protein degradation [23,25,28], to determine whether p62 affects ataxin-3 degradation, we overexpressed or knocked down p62 to assess the protein level of ataxin-3-Q20/Q80. Results showed neither overexpression nor knockdown of p62 affected the protein level of ataxin-3s in HeLa cells (Figure 3A–D), indicating that p62 regulates aggresome formation of ataxin-3-Q80, but has no effect on ataxin-3 turnover.

### 2.4. p62 Promotes the Aggresome Formation of Ataxin-3-80 in a Microtubule-Dependent Manner

**Figure 4:**
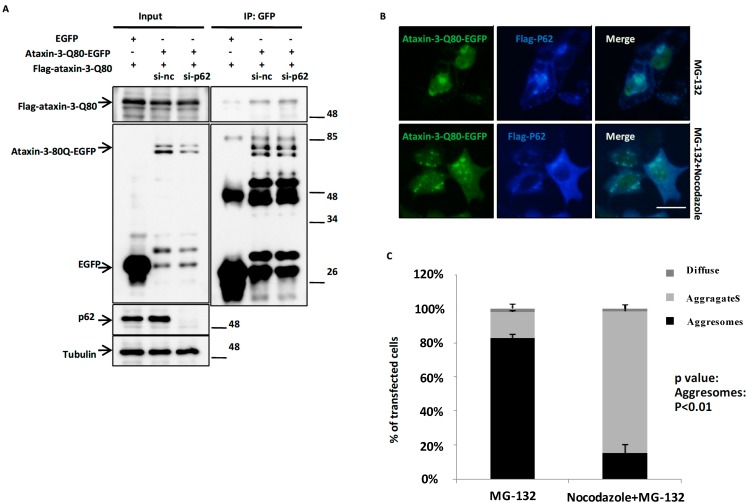
p62 promotes the aggresome formation of ataxin-3-80 depended on microtubule. (**A**) p62 does not affect the self-aggregation of ataxin-3-Q80. H1299 cells were transfected with si-nc or si-p62 siRNA (1# plus 2#) for 12 h, then co-transfected the plasmids of Flag-ataxin-3-Q80 and EGFP or ataxin-3-Q80-EGFP for another 48 h. The cell lysates were subjected to immunoprecipitation assays with anti-GFP antibody. The immunoprecipitants were subjected to immunoblot analysis with the indicated antibodies; (**B**) HEK293 cells were co-transfected the plasmids of Flag-p62 and ataxin-3-Q80-EGFP for 48 h, then treated with MG-132 (10 µM) or MG-132 (10 µM) combined with nocodazole (5 µg/mL) for another 12 h. Then the cells were fixed and subjected to immunofluorescent assay with anti-Flag antibody (Blue). The scale bar indicates 10 µm; (**C**) The quantifications of three different distribution patterns of ataxin-3-Q80-EGFP in (**B**). Three independent experiments were performed and the data are indicated as the means ± S.E.M.

polyQ expanded ataxin-3 is easy to form aggregate in cells due to a self-aggregation tendency [29,30]. To examine whether aggresome formation of ataxin-3-Q80 induced by p62 depends on self-aggregation of ataxin-3-Q80, we knocked endogenous p62 down and performed immunoprecipitation assay. p62 did not regulate the self-association of ataxin-3-Q80 (Figure 4A). The microtubule system is essential for protein transport and to aggresome formation [13,14,15,31,32]. When we de-polymerized microtubule network using nocodazole, the aggresomes of ataxin-3-Q80 disappeared, and were replaced by multiple small aggregates (Figure 4B,C). Meanwhile, p62 still co-localized with ataxin-3-Q80 on those small aggregates (Figure 4B). These observations suggest that p62 targets ataxin-3-Q80 and facilities the microtubule-dependent transport of ataxin-3-Q80 to aggresome.

### 2.5. p62 Protects Cells against Ataxin-3-Q80-Induced Cell Death

**Figure 5:**
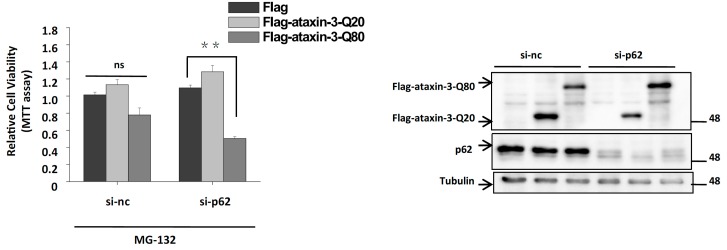
p62 protects cells against ataxin-3-Q80-induced cell death. H1299 cells were transfected with the si-nc or si-p62 siRNA (1# plus 2#) for 12 h, then transfected with the plasmid of Flag, Flag-ataxin-3-Q20, and Flag-ataxin-3-Q80. After 48 h, the cells were treated with MG-132 (10 µM) for 12 h. The cells were subjected to MTT assay to analyze the cell viability. The results are indicated as the means ± S.E.M. ns, no significance; **, *p* < 0.01, one-way ANOVA. The immunoblot showing p62 and ataxin-3-Q20/Q80 expression is presented at the right side. Tubulin served as a loading control.

To further explore the biological function underlying p62-mediated ataxin-3 aggresome formation, we tested ataxin-3–induced cell death in control and p62 depleted cells. Knockdown of p62 significantly increased the cell death rate of ataxin-3-Q80 transfected cells, but not ataxin-3-Q20 transfected cells, indicating that p62 has a protective role against pathogenic ataxin-3-induced toxicity (Figure 5).

### 2.6. Discussion

Although previous studies showed that ataxin-3 could regulate aggresome formation of poly-ubiquitinated proteins, such as mutant CFTR∆F508 and mutant SOD1 [15,33], little is known about its own aggresome formation. Using cultured cell models, we firstly demonstrate that polyQ expanded ataxin-3 could form aggresome. Moreover, we identified p62 as a master regulator of ataxin-3 aggresome formation. p62 has been reported to play a role in the aggregate formation of neurodegenerative disease associated proteins, such as ALS- and Huntington’s disease-associated proteins [23,25]. However, little is known about the role of p62 in aggresome formation. Interestingly, a very recent study showed that p62 is involved in the regulation of aggresome formation of pathogenic prion protein, which is associated with prion disease [24]. Taken together with our observations (Figure 2), we hypothesize that p62 may be broadly involved in the aggresome formation of many neurodegenerative disease associated proteins.

Although it is still not clear whether protein aggregation, the most common feature in neurodegenerative disorders, has a protective or toxic role, growing evidences suggest that the smaller aggregates/oligomers may exert toxic effects and the deposit of these aggregates/oligomers to aggresomes may help cells to maintain homeostasis [18,34,35]. Thus, aggresome formation is usually considered to be a cellular protective mechanism by which the cell handles toxic misfolded protein oligomers or aggregates [36,37]. In agreement with this hypothesis, our results showed a cytoprotective role of p62-mediated aggresome formation of pathogenic ataxin-3 (Figure 5).

A better understanding of the physiological role of ataxin-3 in MJD requires the identification of the specific binding partners of polyQ expanded ataxin-3. Many cell signaling pathway relative proteins, especially the key regulators involved in protein quality control system, have been identified to be associated with pathogenic ataxin-3, such as hHR23A and B, E4B (UFD2a), p45 and VCP/p97 [38,39,40,41,42,43]. In this study, we identified another regulator in protein quality control system, p62, as a novel partner of pathogenic ataxin-3. In our immunoprecipitation experiments, the pathogenic ataxin-3-Q80 immunoprecipitates p62 to a greater extent, compared with wild type ataxin-3-Q20 in cells, (Figure 1C,D). This is consistent with previous studies that pathogenic ataxin-3 shows enhanced binding to its binding partners [40,42]. The association between p62 and ataxin-3 varies *in vitro* and in cells, which shows that p62 does not associate with normal ataxin-3-Q20 *in vitro* but associates with ataxin-3-Q20 in cells, possibly due to the interaction with poly-ubiquitin modified ataxin-3-Q20 in those cells.

Like other ataxin-3 binding partners (such as hHR23A and B, E4B (UFD2a) and VCP/p97), p62 binds poly-ubiquitinated proteins and acts as a shuttling factor in the protein degradation pathways. p62 can regulate the degradation of misfolded tau through the proteasome pathway [28], and degradation of misfolded huntingtin through autophagy pathway [25], thereby playing a protective role. In our observations, the protein levels of ataxin-3-Q20/Q80 were not changed when we overexpressed or knocked down p62 (Figure 3). PolyQ expanded ataxin-3 has a self-aggregation property *in vitro* [29,30]. When we knocked p62 down, the self-association of ataxin-3-Q80 was not affected (Figure 4A). Also, we speculated that p62 could directly associate with ataxin-3-Q80 before targets ataxin-3-Q80 to the aggresomes. As predicted, p62 still co-localized with ataxin-3-Q80 on the small aggregates in the nocodazole treated cells (Figure 4B,C), suggesting that p62 promotes the transport of ataxin-3-Q80 aggregates along microtubule to form aggresome, but is not simply recruited to the aggresomes, or affects ataxin-3 expression and self-aggregation. Furthermore, given the fact that the p62-mediated regulation of autophagy and protein aggregation is tightly coupled with mTOR pathway [44], and recent reports have shown that mTOR signaling pathway is associated with neurodegeneration and oxidative stress [45,46,47], it is possible that p62 cooperates with mTOR signaling to function a role in the regulation of aggresome formation of ataxin-3. Thus, the role of mTOR signaling in p62-mediated aggresome formation of ataxin-3 needs to be further explored.

## 3. Experimental Section

### 3.1. Plasmid Construction

The full-length ataxin-3-Q20/Q80, p62 plasmids were described preciously [41,48]. The PET-21a-p62 was subcloned using EcoR1/Sal sites from a previously described p62-EGFP plasmid [48].

### 3.2. Cell Culture and Transfection

Human embryonic kidney 293 (HEK293) cells, HeLa cells and H1299 cells were cultured in Dulbecco’s modified Eagle’s medium (DMEM) (Gibco, Middleton, WI, USA) containing 10% Fetal Bovine Serum (FBS) (Gibco). For DNA and siRNA transfection, cells were transfected with plasmids or siRNAs using lipofectamine 2000 (Invitrogen, Carlsbad, CA, USA) or Oligofectamine (Invitrogen) respectively. Human siRNA sequences were si-p62: 1# sense: CATGTCCTACGTGAAGGATGAtt, 2# sense; GCATTGAAGTTGATATCGATtt. A negative control (si-nc) was a non-targeting oligonucleotide. Oligonucleotides were purchased from GenePharma (Shanghai, China).

### 3.3. Fluorescent Microscopy

HEK293 Cells were washed with PBS (pH 7.4) and fixed with 4% paraformaldehyde for 10 min at room temperature, then the cells were treated with 0.25% Triton X-100 for 5 min. After blocking with 1% fetal bovine serum for 30 min, cells were incubated with anti-Flag (Sigma, St. Louis, MO, USA), anti-γ-tubulin (Sigma) or p62 (Enzo life science, Farmingdale, NY, USA) antibodies and then with rhodamine (red) or Alexa fluor 350 (Blue) (Invitrogen) conjugated secondary antibody (Santa Cruz Biotechnology, Santa Cruz, CA, USA) for 2 h. The nuclei were stained with DAPI (Sigma). The cells were visualized using an IX71 inverted system microscope (Olympus, Tokyo, Japan).

### 3.4. Immunoblot and Antibodies

Cell extract preparation was used RIPA lysis buffer (25 mM Tris-HCl (pH 7.6), 150 mM NaCl, 1% NP-40, 1% sodium deoxycholate) with the PI (protease inhibitor cocktail) (Roche, Basel, Switzerland). The lysates were separated by 8%–12% SDS-PAGE and transferred onto a PVDF membrane (Millipore, Billerica, MA, USA). Immunoblot analyses were performed using the antibodies: mouse monoclonal antibodies against Flag, Flag-HRP (Sigma), GFP (Santa Cruz), p62/SQSTM1 (Santa Cruz); rabbit polyclonal antibody against GFP [49], p62/SQSTM1 (Enzo life science). The secondary antibodies were sheep anti-mouse IgG-HRP and anti-rabbit IgG-HRP antibodies (Amersham Pharmacia Biotech, Piscataway, NJ, USA). At last, the protein bands were visualized using an ECL detection kit (Amersham Pharmacia Biotech).

### 3.5. Immunoprecipitation

RIPA buffer was used for preparing the cell lysates. The RIPA-insoluble debris was removed after centrifugation at 12,000 rpm for 30 min at 4 °C. The supernatants were subjected to immunoprecipitation with rabbit polyclonal anti-GFP antibody and protein G Sepharose (Roche) for overnight at 4 °C. The protein G sepharose were washed with RIPA buffer six times and then eluted with SDS sample buffer for immunoblot analysis.

### 3.6. GST Pulldown Assay

*In vitro* binding experiment was performed using an aliquot containing approximately 20 µg of GST, GST-ataxin-3-Q20 or GST-ataxin-3-Q80 expressed by *Escherichia coli*, to be incubated with 30 µL of glutathione agarose beads (Pharmacia, Stockholm, Sweden) for 30 min at 4 °C. After washing two times with PBS, the beads were incubated with approximately 40 µg of p62 expressed by *E. coli* BL21 strain for 3 h at 4 °C. At last, the beads were washed five times with the ice-cold PBS. Bound proteins were eluted with SDS loading buffer for immunoblot analysis.

### 3.7. MTT Assay

The transfected cells were washed with DMEM (without phenol red) and incubated with 0.5 mg/mL MTT (3-(4,5)-dim-ethylthiahiazo(-z-y1)-3,5-di-phenytetrazoliumromide) (Sigma) in DMEM. Three hours after incubation, the media were removed and the formazan crystals were dissolved in dimethyl sulfoxide (DMSO). The optical density (OD) was measured by photometer at 570 nm, with background subtraction at 630 nm. The data from three transfection experiments were normalized to control and the ratios were presented.

### 3.8. Statistical Analysis

The western blot densitometry analyses of immunoblots from three independent experiments were performed by photoshop7.0 software (Adobe, San Jose, CA, USA). The data were analyzed using Origin 6.0 (Originlab, Northampton, MA, USA).

## 4. Conclusions

This study includes the following findings: (1) p62 directly associates with ataxin-3-Q80, and co-localizes with ataxin-3-Q80 aggresomes (Figure 1 and Figure 2); (2) p62 regulates ataxin-3-Q80 aggresome formation without affecting the protein level and self-aggregation of ataxin-3-Q80 (Figure 2, Figure 3 and Figure 4); (3) The regulation of ataxin-3-Q80 aggresome formation by p62 has a protective role against ataxin-3-Q80 triggered cell toxicity (Figure 5). Our findings suggest that the p62-ataxin-3-Q80 association could be important in ataxin-3-Q80 aggresome formation and may play roles in MJD pathogenesis.
